# Akathisia Induced by Metoclopramide in a Pregnant Woman With Nausea and Vomiting: A Case Report

**DOI:** 10.1002/npr2.70122

**Published:** 2026-04-14

**Authors:** Ryohei Nakamura, Keisuke Noto, Toshinori Shirata, Yusuke Saito, Isao Takehara, Satoru Nagase, Akihito Suzuki

**Affiliations:** ^1^ Department of Psychiatry Yamagata University School of Medicine Yamagata Japan; ^2^ Department of Obstetrics & Gynecology Yamagata University School of Medicine Yamagata Japan

**Keywords:** adverse drug reaction, akathisia, metoclopramide, perinatal anxiety, pregnancy

## Abstract

**Background:**

Nausea and vomiting are common during pregnancy and can significantly impair maternal well‐being. Metoclopramide is widely used to treat these symptoms but may cause extrapyramidal side effects, including akathisia. Akathisia is characterized by inner‐restlessness and psychomotor agitation, which can be difficult to distinguish from perinatal anxiety disorders due to symptom overlap. Reports of metoclopramide‐induced akathisia during pregnancy are limited.

**Case Presentation:**

We report a case of metoclopramide‐induced akathisia in a pregnant woman at 33 weeks of gestation with nausea and vomiting. She had been taking dextromethorphan for a COVID‐19‐related cough prior to admission. She was hospitalized for threatened preterm labor with abdominal tightness, and intravenous bolus infusions of metoclopramide were initiated for nausea, vomiting, and appetite loss. Shortly thereafter, she developed limb tremors and insomnia, followed by severe inner restlessness and psychomotor agitation. Suspecting anxiety disorders or akathisia, metoclopramide was discontinued and diazepam was started. Her symptoms improved within 3 days, with no recurrence after discontinuation of diazepam. Based on the clinical course and temporal relationship with metoclopramide administration, metoclopramide‐induced akathisia was considered the most likely diagnosis.

**Conclusions:**

This case suggests that metoclopramide‐induced akathisia can occur during pregnancy and may be misdiagnosed as a perinatal anxiety disorder. Rapid intravenous bolus administration is a known risk factor for metoclopramide‐induced akathisia and may have increased the risk in this patient. In addition, a possible pharmacological interaction between metoclopramide and dextromethorphan, including a potential CYP2D6‐related pharmacokinetic effect and central dopaminergic pharmacodynamic effect, may have contributed to akathisia. Greater awareness of this adverse effect may facilitate early recognition and appropriate management.

## Abstract 2

This case report describes a patient who was hospitalized at 33 weeks of gestation and diagnosed with metoclopramide‐induced akathisia. Although the akathisia symptoms resolved 3 days after discontinuation of metoclopramide, a possible interaction with dextromethorphan, which the patient had been orally administering prior to hospitalization, cannot be ruled out.

## Graphical abstract

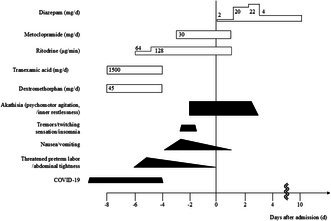

## 1 Background

Nausea and vomiting are common conditions during pregnancy, affecting up to 70%–80% of pregnant women, and can significantly affect maternal well‐being. The most severe form, hyperemesis gravidarum, occurs in approximately 0.3%–3% of pregnancies and is associated with significant weight loss, dehydration, and electrolyte imbalance [1]. Metoclopramide is an antiemetic agent commonly used to manage nausea and vomiting during pregnancy, including hyperemesis gravidarum [1]. Metoclopramide easily crosses the blood—brain barrier [2], and exerts its antiemetic effects by antagonizing dopamine D2 and serotonin 5‐HT3 receptors in the chemoreceptor trigger zone of the area postrema [3]. However, this drug may also cause extrapyramidal side effects such as akathisia, akinesia, and dystonia [4, 5, 6]. Among these side effects, akathisia is characterized by both subjective symptoms, including feelings of restlessness and inner tension, and objective symptoms, including purposeless leg movements and an inability to sit still [7]. The incidence of akathisia has been reported to be 10%–20% among patients treated with metoclopramide [8], and the discomfort caused by metoclopramide‐induced akathisia has been reported to lead to anxiety disorders, depression, or suicidal attempts [9, 10].

The perinatal period is recognized as a time of increased vulnerability to psychiatric disorders, particularly anxiety disorders [11]. Notably, the symptoms of perinatal anxiety often overlap with the subjective features of akathisia, making differential diagnosis difficult [8]. Despite the widespread use of metoclopramide, to the best of our knowledge, only three previous cases of metoclopramide‐induced akathisia during pregnancy have been reported [12, 13, 14]. We report a case of metoclopramide‐induced akathisia in a pregnant woman, in whom distinguishing drug‐induced akathisia from perinatal anxiety disorder was clinically challenging. In this context, the present report is intended mainly to raise clinical awareness of this adverse effect in pregnant patients.

## 2 Case Presentation

The subject was a woman in her 20s with no history of psychiatric illness or illicit drug use. She was a nonsmoker and did not consume alcohol. Her pregnancy had been uneventful and was considered normal. During the pregnancy, she had not exhibited any symptoms of perinatal anxiety or depression. She reported no financial or marital concerns and received sufficient emotional and physical support from her family. This report complies with the Declaration of Helsinki and was approved by the Ethical Review Committee of the Yamagata University Faculty of Medicine. Written informed consent was obtained from the patient for publication.

**FIGURE 1:**
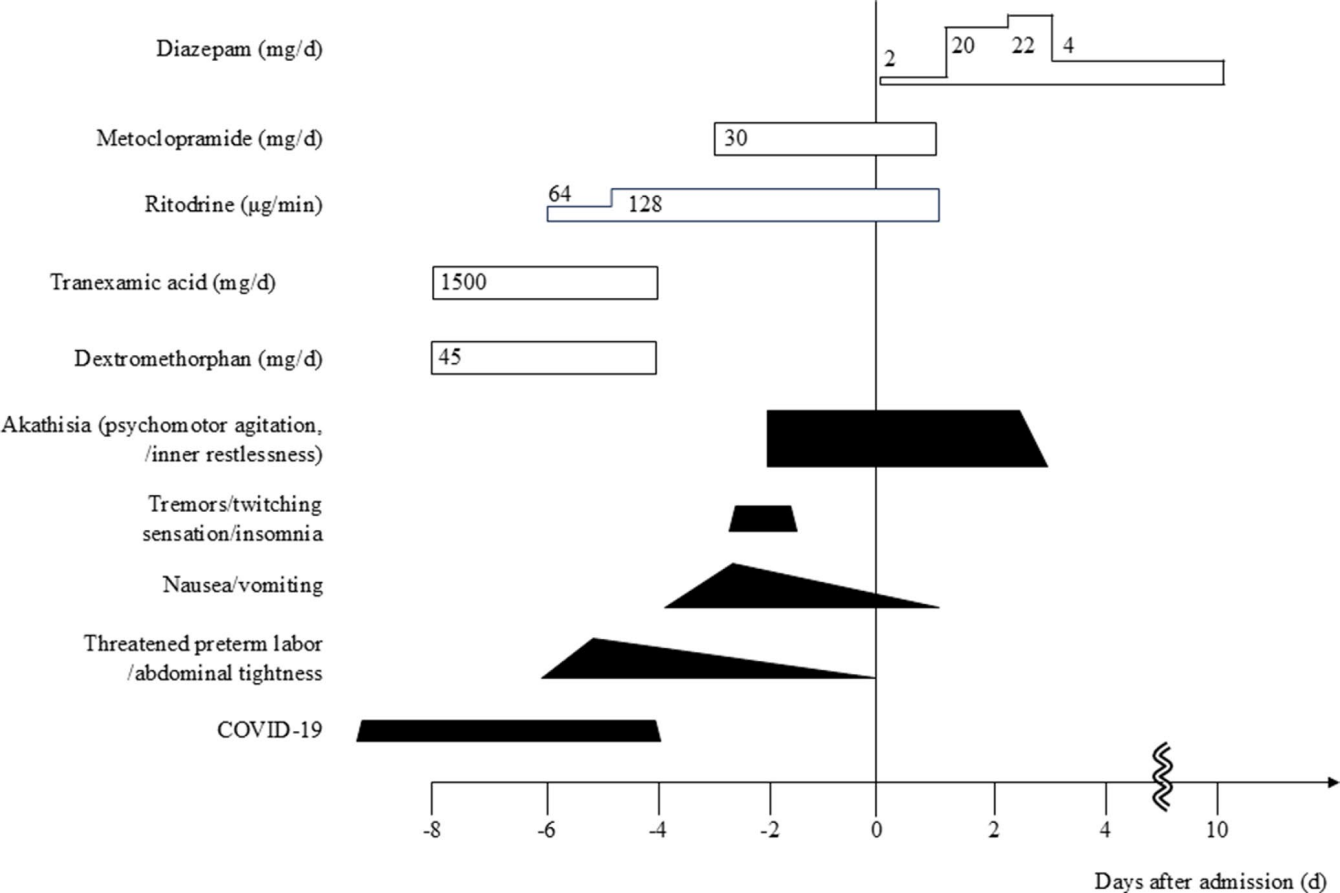
Timeline of the clinical course of the patient.

Figure 1 shows the timeline of the clinical course. Eight days before admission to our hospital (32 weeks of gestation), she was diagnosed with COVID‐19 and treated symptomatically with dextromethorphan (45 mg/day) for cough and tranexamic acid (1500 mg/day) for sore throat at a clinic. Six days before admission (33 weeks of gestation), she was hospitalized at a hospital for threatened preterm labor and abdominal tightness, and treated with ritodrine (65–130 μg/min). Four days prior, dextromethorphan and tranexamic acid were discontinued. As nausea, vomiting, and loss of appetite emerged and worsened, intravenous bolus infusion of metoclopramide (30 mg/day, in three divided doses) was initiated 3 days before admission. Two days prior to admission, she developed tremors predominantly in the right limbs, a twitching sensation, and insomnia. Neurological examination by a neurologist revealed no abnormalities, and she was kept under observation. Subsequently, she reported inner restlessness along with a sense of being unable to stay still throughout the day, describing feelings such as “I can't settle down,” “I can't sit still,” and “I feel like I have to go somewhere.” These symptoms were not alleviated by walking. She frequently got in and out of bed and paced around her room, exhibiting marked psychomotor agitation. She was transferred to our hospital.

On admission, in addition to the subjective inner sense of restlessness and anxiety, objective signs of psychomotor agitation, including fine whole‐body movements, repeated standing and sitting, palmar sweating, and tachypnea were observed. No other neuropsychiatric symptoms such as hallucinations, delusions, thought disturbance, and altered consciousness were found. Blood tests, including thyroid function and infection screening, were unremarkable. Serum iron and folate levels were not measured in this case. Brain magnetic resonance imaging (MRI), including diffusion‐weighted imaging and fluid‐attenuated inversion recovery, showed no abnormalities. Due to suspicion of either an anxiety disorder or akathisia, metoclopramide and ritodrine were discontinued. Oral diazepam was started at 8–22 mg/day.

Her psychiatric symptoms improved by the third day after admission. She was discharged on the eighth day after admission. Diazepam was discontinued on the 10th day, and no recurrence of psychiatric symptoms was observed thereafter.

## 3 Discussion

Because the patient was pregnant, invasive procedures such as lumbar puncture and whole‐body computed tomography were not performed. However, there were no signs of inflammation on blood testing, and brain MRI revealed no abnormalities; therefore, COVID‐19 encephalitis or other encephalitis was considered unlikely. She had no history of alcohol abuse or illicit drug use, and thus psychiatric symptoms due to such substances were also ruled out. It has been suggested that the subjective symptoms of akathisia are difficult to distinguish from perinatal‐onset psychiatric disorders such as anxiety disorder, depression, and bipolar disorder [8]. However, Sachdev [7] proposed research diagnostic criteria for drug‐induced akathisia that require exposure to a causative drug, the presence of characteristic subjective and objective features, and the exclusion of other known causes. In addition, a definite diagnosis requires at least one subjective feature and one objective feature to be present on two examinations separated by at least 1 day. In the present case, the patient developed a subjective feeling of inner restlessness together with observable restless movements after the initiation of metoclopramide, and no alternative neurological or substance‐related cause was identified. Thus, her presentation was clinically consistent with acute metoclopramide‐induced akathisia according to Sachdev's criteria. Furthermore, psychiatric symptoms appeared 1 day after the initiation of metoclopramide and improved within 3 days after its discontinuation. Extrapyramidal symptoms such as tremors were also observed during this period. In addition, the total duration of psychiatric symptoms was 6 days, which did not meet the diagnostic criteria for a primary psychiatric disorder according to DSM‐5 [15]. No recurrence of symptoms was observed after discontinuation of anxiolytic treatment. Taken together, these findings suggest that the symptoms in this case were attributable to metoclopramide‐induced akathisia.

The incidence of akathisia induced by metoclopramide has been estimated at 10%–20% of patients [8], and it is suggested that pregnant women are further at high risk for the development of medication‐induced akathisia due to hormonal fluctuations such as estrogen, psychosocial stressors associated with pregnancy, and deficiencies in serum iron or folate [16]. However, despite the widespread use of metoclopramide for nausea and vomiting during pregnancy [1], to the best of our knowledge, only three previous cases of metoclopramide‐induced akathisia in pregnant women have been reported [12, 13, 14]. This paucity of reports suggests that metoclopramide‐induced akathisia during pregnancy may be underrecognized or misdiagnosed as another psychiatric disorder. Therefore, the main clinical value of the present case is not novelty of mechanism, but reinforcement of awareness that this adverse effect may occur during pregnancy and may be confused with perinatal anxiety symptoms.

From a practical clinical perspective, the most difficult point at the initial assessment is that both perinatal anxiety and akathisia can present with restlessness, insomnia, anxiety, and inability to remain calm. In such situations, forward‐looking clinical judgment should rely not only on symptom description but also on several bedside clues. First, careful review of recent medication exposure is essential, particularly the initiation or dose escalation of dopamine antagonists. Second, the presence of observable motor signs, such as repeated standing and sitting, pacing, or inability to remain seated, may support akathisia rather than primary anxiety. Third, abrupt onset within a short period after drug administration may also favor drug‐induced akathisia. Finally, close short‐term follow‐up after discontinuation of the suspected drug is important because improvement after withdrawal may retrospectively strengthen the diagnosis. In actual practice, however, these distinctions are not always clear at presentation, especially in pregnant patients, and akathisia should be included in the differential diagnosis when new restlessness develops after antiemetic treatment.

The present patient received metoclopramide via intravenous rapid bolus infusions. There are several studies that have examined the relationship between the incidence of akathisia and the intravenous infusion rate of metoclopramide in patients with nausea, vomiting, or headache [5, 17, 18]. These studies suggest that subjects who received a rapid intravenous bolus of 10 mg metoclopramide over 2 min had a significantly higher incidence of akathisia compared with those who received the same dose infused over more than 15 min, despite no significant difference in antiemetic efficacy between the two groups. Therefore, rapid bolus administration should be regarded as a known clinical risk factor in this case, rather than as a distinctive feature of the present report.

Bateman et al. [4, 19] suggested that akathisia occurs when peak plasma concentrations of metoclopramide exceed 100–120 ng/mL. It has been shown that most of metoclopramide absorbed into the body is metabolized to monodeethylmetoclopramide by hepatic cytochrome P450 (CYP) enzymes, with this metabolism being primarily mediated by CYP2D6, and to a lesser extent by CYP3A4 and CYP1A2 [3, 20]. Meanwhile, in the present case, dextromethorphan had been administered until the day just before metoclopramide initiation. Dextromethorphan is known to be a substrate of CYP2D6 [3] and has been reported to potentially act as a competitive inhibitor of CYP2D6 [21, 22]. Thus, a CYP2D6‐related pharmacokinetic interaction can be considered as a background pharmacological possibility. Meanwhile, dextromethorphan has been reported to exhibit serotonin reuptake inhibitory properties and to promote serotonin release [23]. Selective serotonin reuptake inhibitors such as escitalopram [24] and fluoxetine [25] have also been implicated in inducing akathisia in depressed patients, possibly via serotonin‐mediated inhibition of the dopaminergic system within the mesocorticolimbic pathway projecting from the ventral tegmental area to the prefrontal cortex [24]. Therefore, a pharmacodynamic interaction involving the central dopaminergic system may also be considered as a possible contributing factor. However, these pharmacokinetic and pharmacodynamic mechanisms remain speculative.

This case report has several limitations. First, we did not measure plasma concentrations of metoclopramide or evaluate CYP2D6 genotype or phenotype; therefore, the proposed pharmacokinetic mechanism could not be confirmed. Second, invasive testing, for example, cerebrospinal fluid examination, was avoided due to pregnancy, which might have helped exclude other neurological conditions. Third, symptom severity and its temporal change were not assessed quantitatively using a validated rating scale such as the Barnes Akathisia Rating Scale [26]. Therefore, the degree of improvement after discontinuation of metoclopramide and initiation of diazepam should be interpreted cautiously. Finally, as this is a single case report, the findings cannot be generalized, and further accumulation of similar cases is necessary to clarify the relationship between metoclopramide use during pregnancy and the development of akathisia.

## 4 Conclusions

The present case suggests that administration of metoclopramide can induce akathisia during pregnancy, and this condition may be misdiagnosed as perinatal anxiety disorders. In this patient, rapid intravenous bolus administration is a known risk factor for metoclopramide‐induced akathisia and may have increased the risk of akathisia. In addition, a possible pharmacological interaction between metoclopramide and dextromethorphan, including a potential CYP2D6‐related pharmacokinetic effect and a central dopaminergic pharmacodynamic effect, may have contributed to akathisia. The main contribution of this report is to increase clinical awareness of metoclopramide‐induced akathisia during pregnancy and to emphasize the need for careful differential diagnosis from perinatal anxiety symptoms.

## Author Contributions

K.N. and A.S. conducted inpatient care management of the patient. R.N., T.S., and A.S. wrote the draft of the manuscript. Y.S., I.T., and S.N. supervised the treatment, and provided detailed comments and revisions on subsequent drafts. All the authors have read and approved the final manuscript.

## Funding

The authors have nothing to report.

## Ethics Statement

This report was approved by the Ethical Review Committee of the Yamagata University Faculty of Medicine.

## Consent

Written informed consent was obtained from the patient for the publication of this case report.

## Conflicts of Interest

The authors declare no conflicts of interest.

## Data Availability

Data sharing is not applicable to this article as no datasets were generated or analyzed during the current study.
